# Evaluation of retinal structure changes with AI-based OCT image segmentation for sodium iodate induced retinal degeneration

**DOI:** 10.3389/fncel.2025.1605639

**Published:** 2025-06-18

**Authors:** Yong Zeng, Jiaming Zhou, Yichao Li, Bruno Alvisio, Jacob Czech, David Bissig, Haohua Qian

**Affiliations:** ^1^Visual Function Core, National Eye Institute, National Institutes of Health, Bethesda, MD, United States; ^2^Northwestern University, Evanston, IL, United States; ^3^OSIO Bioinformatics Core, National Eye Institute, National Institutes of Health, Bethesda, MD, United States; ^4^Department of Neurology, University of California, Davis, Sacramento, CA, United States

**Keywords:** mouse, retina, optical coherence tomography, sodium iodate, segmentation, choroid, biomarker, artificial intelligence

## Abstract

Segmentations of retinal optical coherence tomography (OCT) images provide valuable information about each specific retinal layer. However, processing images from degenerative retina remains challenging. This study developed artificial intelligence (AI)-based segmentation to analyze structure changes in sodium iodate (SI)-treated mice. The software is capable of segmenting seven retinal layers and one choroid layer. Analyzing OCT images captured at days post SI-injection (PI) revealed early changes in the retinal pigment epithelium (RPE) layer, with increase in thickness and reduction in reflectance calculated by estimated Attenuation Coefficients (eAC). On the other hand, eAC for outer nuclear layer (ONL) exhibited early and sustained increase after SI treatment. SI induced exponential reduction in ONL thickness with a half-reduction time of about 3 days, indicating progressive photoreceptor degeneration. The extent of degeneration was correlated with ONL eAC level at PI1. Inner retinal layers showed bi-phasic reactions, with initial increases in layer thickness that peaked at around PI3, followed by gradual reduction to lower than baseline levels. In addition, SI also induced transient increases in vitreous particles concentrated around the optic nerve head. Furthermore, there was a gradual reduction of choroid thickness after SI treatment. These results indicate the AI-segmentation tool's usefulness for providing a sensitive and accurate assessment of structure changes in diseased retina and revealed more detailed characterization of SI-induced degeneration in all retinal layers with distinct time courses. Our results also support ONL reflectance changes as an early biomarker for retinal degeneration.

## 1 Introduction

Optical Coherence Tomography (OCT) is a well-developed non-invasive imaging technology that provides depth-penetrating images of living biological tissue, including the retina (Fischer et al., 2009; Kubo and Akasaka, 2010; Vinekar et al., 2010; Baghaie et al., 2015). Differences in tissue scattering and reflectivity provide image contrast. Retinal images are thereby segmented to reveal vital information about the retinal structure, layer thickness, and reflective intensity (Fischer et al., 2009; Genead et al., 2011; Baghaie et al., 2015; Zeng et al., 2016; Tatham and Medeiros, 2017; Zeng et al., 2024). OCT is widely used to clinically track conditions like macular degeneration (AMD), diabetic retinopathy (DR), glaucoma retina, and inherited retinal diseases in human and animal models (Spaide, 2013; Kwan and Fawzi, 2019; Ling et al., 2020; Tong et al., 2023) because abnormalities in retinal layer thickness or OCT reflectivity imply disease. These specific findings can reveal clues about disease progression and underlying mechanisms (Furashova and Matthe, 2017; Bannai et al., 2020; Gersch et al., 2022; Moraes et al., 2024; Riazi-Esfahani et al., 2024). Improvements in measurement accuracy for each layer's thickness and reflectivity will presumably enhance the clinical and basic science value of OCT images.

Historically, quantitative analysis of retinal OCT images relied on labor-consuming manual measurements, or explicitly defining features of specific retinal layers within a software package, automating some of the work. The latter is often frustrated by diseases of the retina, precisely because of the OCT abnormalities they cause. Recent advancements in artificial intelligence (AI) allow for automated measurement with greater flexibility, offering a standardized and objective method of quantifying abnormalities in retinal disease (Kwan and Fawzi, 2019; Schmidt-Erfurth et al., 2020; Keenan et al., 2021; Yang et al., 2021). AI-based programs are particularly useful in field of ophthalmology, with multi-modulatory imaging data unveiling new clinical and pathogenic insights into retinal diseases (Lee et al., 2017; Lu et al., 2019; Narendra Rao et al., 2019; Schmidt-Erfurth et al., 2020; Mares et al., 2024). In this study, we adapted an AI-based segmentation program to OCT images of the degenerated retina from a mouse model.

Sodium iodate (NaIO_3_, SI), a chemical known to induce retinal cell death via oxidative stress, is commonly used to produce retinal degenerative animal models. Both intraperitoneal and intravenous applications of SI induce dose-dependent retinal degeneration in various animal models (Ou et al., 2018; Koh et al., 2019; Ahn et al., 2020; Koster et al., 2022). It is well established that SI first impacts retinal pigment epithelium (RPE) activity (Noell, 1953, 1954; Moriguchi et al., 2018; Liu et al., 2019). With SI-induced retinal degeneration monitored by OCT imaging, we hypothesize that accurate segmentation of the retinal layers with the AI-based program will not only reveal expected damage to the RPE and thinning of the photoreceptor layer but also uncover novel effects of NaIO_3_ in all retinal layers. In this study, we monitored retinal structure changes during the time course of degeneration in mice that received an intraperitoneal injection of SI with OCT imaging. Using the AI-based segmentation program, we segmented OCT images captured at different stages of diseased retinas following several timepoints post-SI injection. Information about each retinal layer's thickness and reflectance intensity were analyzed to characterize retinal degeneration in the mouse model. To quantify reflectance changes in the retinal layers, we calculated estimated Attenuation Coefficient (eAC; Vermeer et al., 2013; Moriguchi et al., 2018; Chang and Bowden, 2019; Sakai et al., 2024; Bissig et al., 2025), as raw OCT intensity is influenced by many factors, including the optical quality of the cornea and anterior chamber. eAC was used to correlate early changes in ONL reflectance with the extent of photoreceptor degeneration. Furthermore, we also explored the thickness of the inner retina and choroid layer with our AI-based segmentation program—and opportunity to provide additional sensitive and unbiased layer-specific metrics, including the first quantitative analysis of choroid thickness for SI-induced retinal degeneration.

## 2 Methods and materials

### 2.1 Animal

This research was conducted in accordance with the ARVO Statement for the Use of Animals in Ophthalmic and Vision Research and was approved by the Animal Care and Use Committee of the National Eye Institute. C57BL/6J mice were reared under 50-104 lux cyclic lighting (12 h:12 h) with food and water available ad libitum. Seven 2-month-old male C57BL/6J mice were intraperitoneally injected with 25 mg/kg of NaIO_3_. The size of animal used in this study is typical for investigating SI-induced retinal degenerations (Arifin and Zahiruddin, 2017; Moriguchi et al., 2018; Kim et al., 2022).

### 2.2 OCT image collection and image processing

OCT images were collected from both eyes of the animals before (baseline) and 1, 3, 6, 13, and 20 days post-SI injection. Prior to imaging, the animals were anesthetized with xylazine 6 mg/kg and ketamine 100 mg/kg. Drops of tropicamide (1%) and phenylephrine (2.5%) were then topically applied to the eyes for pupil dilation. OCT images of the mice were obtained with Bioptigen's Envisu R2210 system (axial resolution 1.7 μm/pixel), using a protocol for 4 B-scans of the retina (1.4mm) at 45-degree intervals with each B-scan sampled 40 times. Averaged Bscan images (4 per eye) were fed to AI models for retinal layer segmentation. A total of 112 image sets were analyzed at each time points.

eAC were calculated based on published methods (Vermeer et al., 2013; Bissig et al., 2025). Briefly, OCT b-scans were transformed so that grayscale pixel values were linearly proportional to the power of the signal measured by the device. In each A-scan, attenuation is described by pixel size, the brightness of a pixel, and the relative brightness of remaining pixels more distant from the OCT device. In our commercial system, there was very little variance in retinal distance from the OCT sensor so the signal decay factor was ignored in these calculations [using the variable names of Vermeer et al. (2013), we stipulate I(z) = U(z)-N(z)]. Acknowledging this slight change from Vermeer et al. and the existence of other strategies to calculate the attenuation coefficient (Chang and Bowden, 2019), we refer to our results as *estimated* attenuation coefficients (eAC) with units of m^−1^ and expressed on a logarithmic scale.

The vitreous portion of the image was identified as everything above the first segmentation line (boundary of inner limiting membrane with vitreous). Particles in vitreous were determined using the “Analyze Particles” function in ImageJ. A size range of 5 to 500 was selected to exclude small artifacts and the large optic nerve head in images.

### 2.3 AI-based OCT segmentation

The program for building of machine learning models were described previously (Berkowitz et al., 2022). Briefly, the model was based on a U-net convolutional neural network trained using the “dice loss” function and the Adam optimizer (learning rate = 0.001). A shortest-path algorithm was applied to improve the model's performance. Using “U-net” architecture, five models were built from annotated OCT images with training parameters varied in the number of convolution layers, kernel, starting neurons, and pool layers. Five attentional models were built with the same training parameters but from annotated OCT images pre-processed using a contrast-enhancing algorithm (Girard et al., 2011). Furthermore, two additional models were made with “deeplabv3plus” architecture. A custom-made MATLAB program displayed OCT images superimposed with the segmentation lines generated by each model, along with a segmentation line based on the median values of selected models. The accuracy of each segmentation line was verified manually by either selecting best fit from model-predicted pools or making manual corrections using Pisces2D (v2.1.8342, Voxeleron). As OCT images were captured with the optic nerve head centered, segmentations on either side of the optic nerve head were independently evaluated.

### 2.4 Data analysis

For each B-scan, two-200-pixel (A-scan) region (350 to 630 um away from center of ON head) were selected for measurement (Gao et al., 2021). Unless specified otherwise, averaged values from 8 ROIs were used to represent each eye and data are presented as mean ± SEM. Paired *t*-tests were used to check for significance of change from the baseline values. Statistics were performed with Prism (V10.2.3, GraphPad software).

## 3 Results

### 3.1 AI-models and retina segmentation

**Figure 1:**
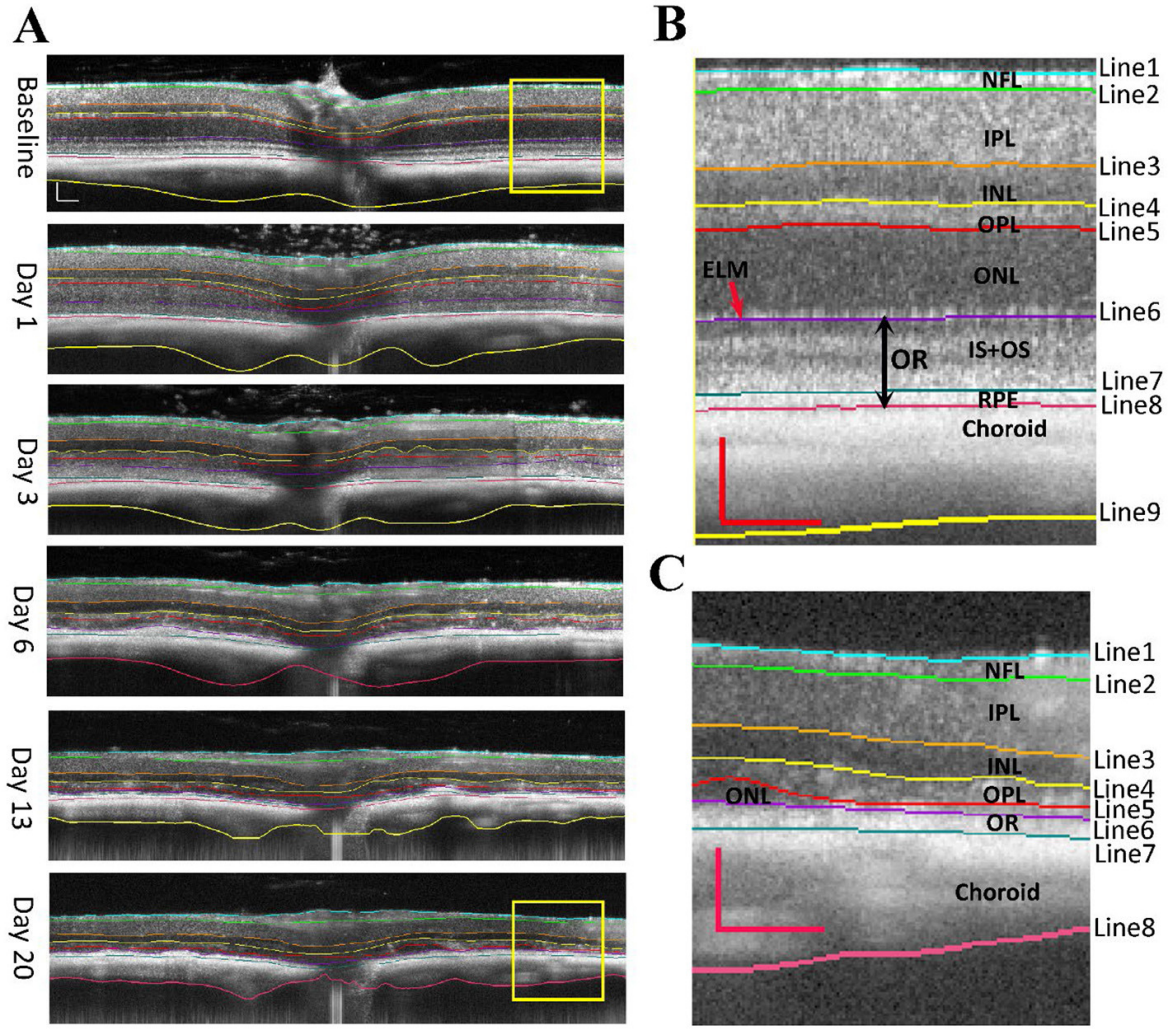
OCT images of NaIO3 -induced retinal degeneration with segmented lines. **(A)** longitudinal scans of the same mouse eye before (baseline) and at day 1, day 3, day 6, day 13, and day 20 of post injection (PI). **(B)** Magnified view of baseline image with segmentation. **(C)** Magnified view of Day 20 image with segmentation. Segmented layers are labeled: NFL nerve fiber layer, IPL inner plexiform layer, INL inner nuclear layer, OPL outer plexiform layer, ONL outer nuclear layer, OR outer retina (ELM external limiting membrane, IS + OS photoreceptor cell inner and outer segments, and RPE retinal pigment epithelium). Scale bar: 100 μm.

**Figure 2:**
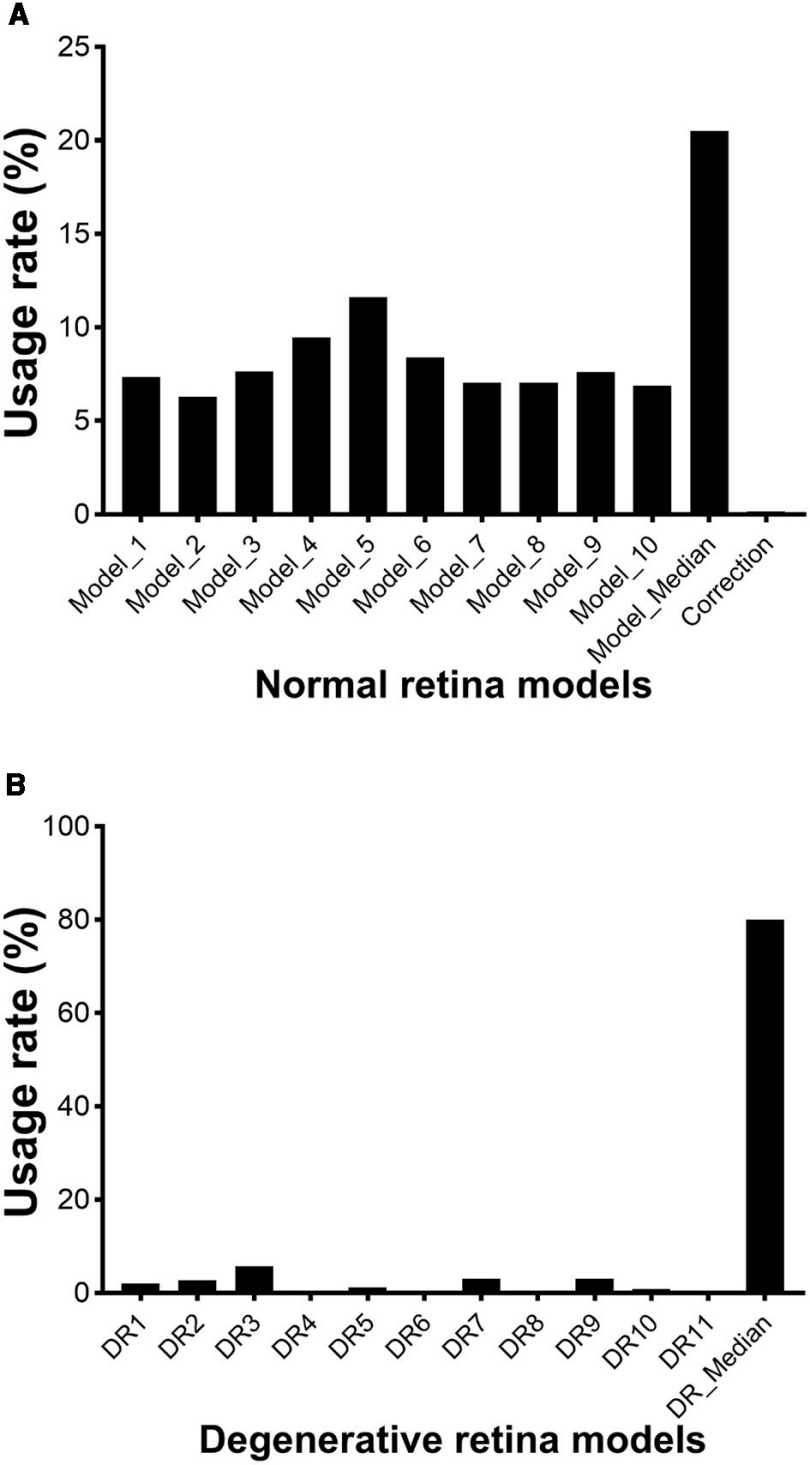
Usage rate of each AI-generated model for segmenting OCT images of mouse retina. **(A)** normal retinal models; **(B)** degenerative retina models. Lines starts from vitreoretinal boundary, and the last line represent the end of choroid. L: left-field, R: right-field of OCT image with optic nerve head placed in the center.

Figure 1A shows examples of retinal OCT images captured from the same eye at indicated time points before and after SI treatment. We generated 10 “U-net” models (normal retina models) based on annotated OCT images previously captured from normal mouse retinas. These models segment OCT images with nine lines to produce seven retinal layers and a choroid layer (Figure 1B). The normal retina models can successfully segment OCT images captured at baseline, PI1, and PI3, with very few (< 0.2%) manual corrections needed. To compare each model, we calculated usage rate, ratio of image user selected as the best fit vs. total number of images analyzed. Usage rates for each normal retina model are summarized in Figure 2A (with details listed in Supplementary Table 1 and DICE score listed in Supplementary Table 2). We also calculated the median point for every A-scan from values 10 models predicted and called “Model_median”, which had the highest usage. On the other hand, each of 10 individual models had its own success rate indicating all models are useful in predicting segmentation lines.

However, normal retina models performed poorly for images captured at PI6, PI13, and PI20 when retinal degeneration becomes imminent. We generated 11 models using both “U-net” and “deeplabv3plus” architectures (degenerative retina models). Training dataset includes annotated OCT images previously captured from rd10 mice and images from normal retina with the photoreceptor layer artificially reduced. These models contain 8 segmentation lines to produce 6 retinal layers and a choroid layer (Figure 1C). Usage rates for these models in analyzing images captured at PI6, PI13, and PI20 are summarized in Figure 2B (with details listed in Supplementary Table 3 and DICE score listed in Supplementary Table 4). The median point for every A-scan from values 11 models predicted and called “DR_median”, which also had the highest success rate, whereas all individual “degenerative retina” models were useful in predicting segmentation lines. All OCT images were able to be analyzed with existing models and no manual correction was needed.

### 3.2 Segmentation of retinal and choroid layers

Example segmentation lines are superimposed on OCT images in Figure 1A. Images from baseline, PI1, and PI3 are segmented with “normal retina” model, which uses nine segment lines to separate an image into eight layers: the nerve fiber layer (NFL), inner plexiform layer (IPL), inner nuclear layer (INL), outer plexiform layer (OPL), outer nuclear layer (ONL), inner and outer segment layer (IS + OS), retinal pigment epithelium (RPE) and choroid layer. The magnified view of a baseline OCT image outlined by a yellow box is shown in Figure 1B. It better-illustrates the placement of the nine lines. Starting at PI6, retinal degeneration became more extensive, illustrated by thinning of photoreceptor layers, merging of photoreceptor and RPE layers, and reduced prominence of the external limiting membrane (ELM). OCT images obtained from these injured retinas could not be adequately processed with “normal retina” model. We developed “degenerative retina” model to analyze these images, with eight segmentation lines to separate seven layers. As the IS + OS and RPE layers merged, we used the outer retina (OR) to define this merged layer. A magnified image of the region outlined by a yellow box for PI20 OCT is shown in Figure 1C.

### 3.3 Effect of NaIO_3_ on RPE and photoreceptor layers

**Figure 3:**
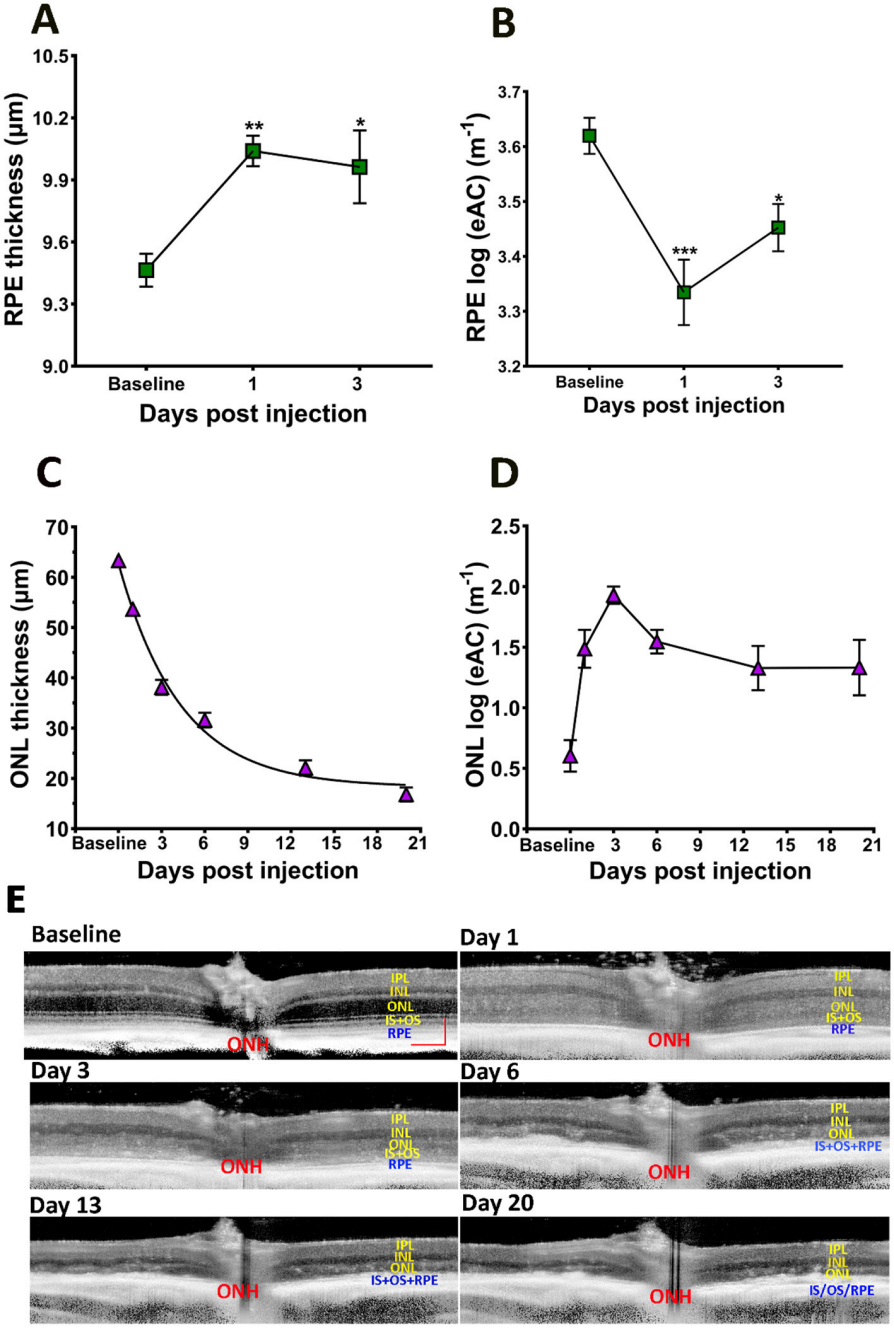
Effects of SI on RPE and photoreceptor layers. **(A)** RPE layer thickness. **(B)** RPE layer eAC. **(C)** ONL thickness. **(D)** ONL eAC. **(E)** Examples of eAC derived from OCT images. Scale bar: 100 μm.

Using the “normal retina” model, the RPE layer can be separated from OCT images captured from baseline, PI1, and PI3 eyes. As shown in Figure 3A, there is a significant increase in RPE layer thickness after SI injection for both PI1 and PI3, consistent with swelling of RPE cells in response to SI. We used estimated Attenuation Coefficient (eAC) to probe SI induced reflectance changes on the RPE layer (Vermeer et al., 2013; Moriguchi et al., 2018; Chang and Bowden, 2019; Sakai et al., 2024). Examples of eAC images calculated from the original OCT images are shown in Figure 3E. The effects of SI treatment on RPE layer reflectivity are summarized in Figure 3B. There is a significant reduction of reflectance in RPE cells after SI application.

**Figure 4:**
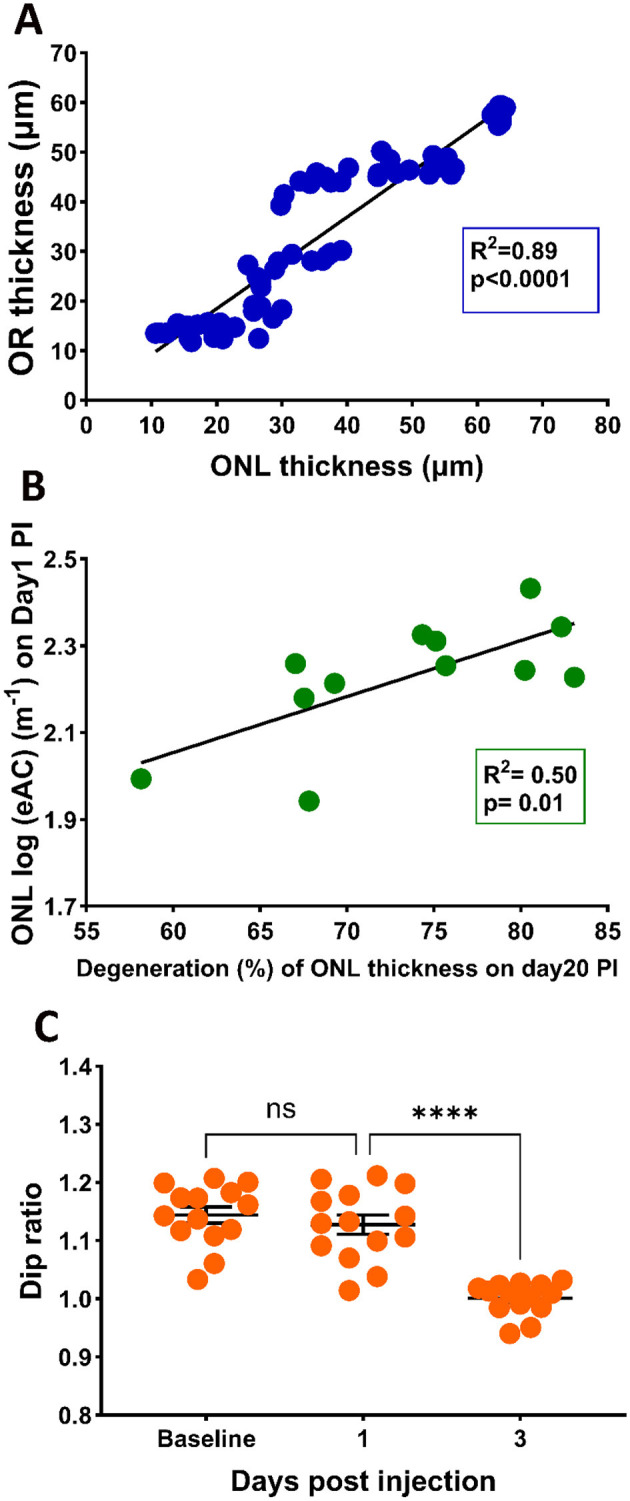
**(A)** Correlation between ONL thickness and OR thickness **(A)** with the 95% confidence interval for R^2^ of 0.91 to 0.96. **(B)** Correlation between photoreceptor degeneration (ONL reduction at PI20) and ONL eAC values at PI1 **(B)** with the 95% confidential interval of R^2^ of 0.22 to 0.91, **(C)** Effects of SI on Dip ratio, defined as OCT intensity at IS peak divided by the value at Dip region.

SI is known to induce photoreceptor degeneration. Figure 3C plots ONL thickness during the course of the study, which showed gradual reduction after SI application. The time course of photoreceptor degeneration can fit with an exponential decay with a half-decay time of ~3 days. Application of SI also changed reflectivity of the ONL layer: there, eAC values are visibly elevated on PI1, and this elevated level of reflectivity is maintained thereafter (Figure 3). Photoreceptor degeneration reduces thickness for both ONL and OR. Consequently, there is a high correlation between ONL thickness and OR thickness following application of SI, as shown in Figure 4A.

### 3.4 Correlation of reflectance elevation with retinal degeneration

We hypothesized that elevated reflectance for the ONL layer could serve as an indication of photoreceptor stress. To test this hypothesis, we calculated the correlation of the eAC values of ONL observed on PI1 and the extent of photoreceptor degeneration at PI20. Results are shown in Figure 4B. A linear fit of the data indicates good correlation (*R*^2^ = 0.50) between the eAC on P-I1 and amount of ONL reduction on PI-20, suggesting that ONL reflectance could serve as an early marker for photoreceptor degeneration.

It has been suggested that the Dip ratio (OCT intensity of IS band divided by the intensity of the hyporeflective zone between IS and OS, illustrated in Supplementary Figure 1) could serve as an early biomarker for retinal degeneration (Zeng et al., 2024). Figure 4C presents the Dip ratio calculated from OCT images captured at baseline, PI-1 and PI-3. Compared with baseline levels, a significant reduction in Dip ratio was observed at the PI-3 time point, providing additional support that Dip ratios could serve as a biomarker for retinal degeneration. On the other hand, there is no significant different in the Dip ratios between PI-1 and baseline, suggesting this biomarker lags ONL reflectivity in predicting SI-induced degeneration.

### 3.5 Effect of SI on inner retina

**Figure 5:**
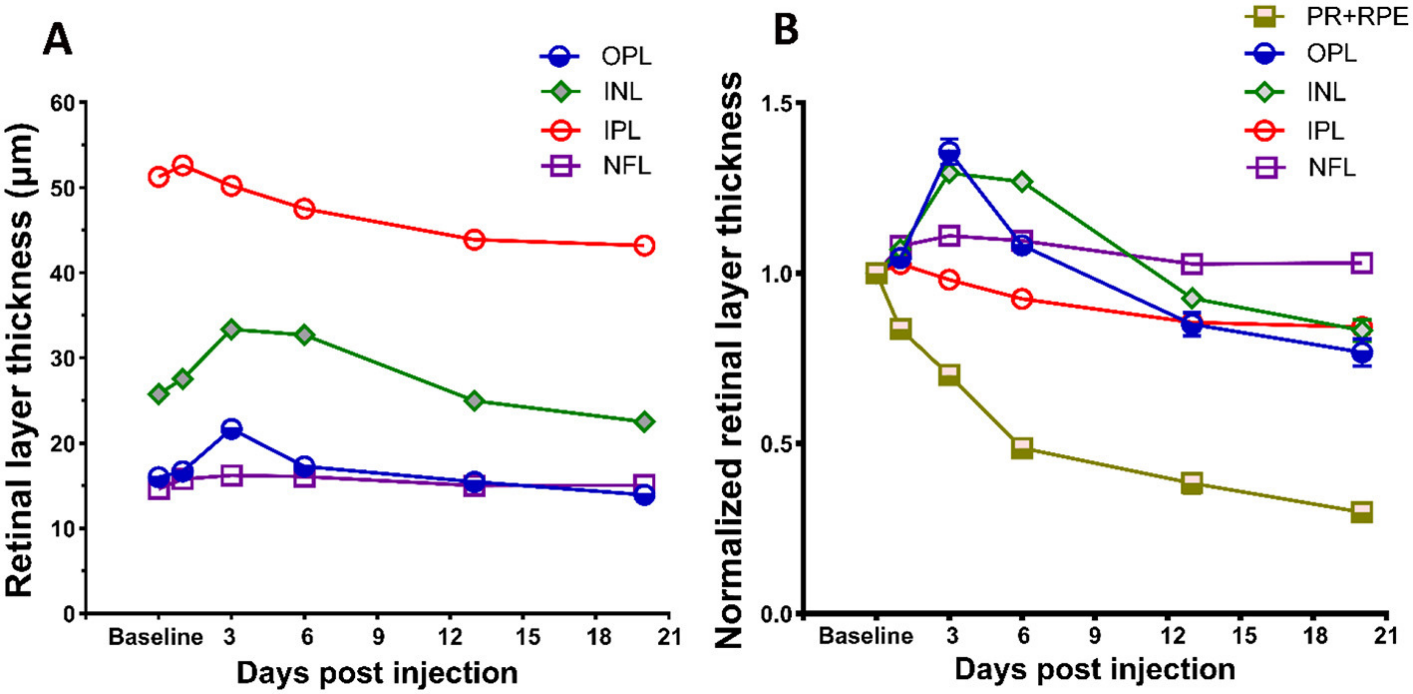
Effects of SI on inner retinal layers. **(A)** SI induced layer thickness changes for four inner retina layers. **(B)** Layer thickness normalized to their respective baseline value. Thickness of photoreceptor and RPE complex (PR + RPE) is also included for comparison with four inner retinal layers. Error bars represent SEM (unless smaller than the size of the symbols).

In addition to alterations in photoreceptor and RPE layers, application of SI also elicited responses in the inner retinal layers. Changes in inner retinal layer thicknesses are shown in Figure 5A, and the thicknesses of each layer normalized to baseline value are shown in Figure 5B. There is an increase of inner retinal layer thicknesses after SI injection, suggesting swelling of all inner retinal layers. Maximum swelling was observed at PI3, with OPL and INL exhibiting large changes as shown on the normalized plot (Figure 5B). This swelling slowly regresses with time, and at PI20 most inner retinal layers (except NFL) showed a significant reduction of layer thickness than baseline. Table 1 summarizes retinal layer thicknesses at baseline and post-injection changes.

**Table 1:** Retinal layer thickness at baseline and post SI treatment (mean ± SD in μm).

	**NFL**	**IPL**	**INL**	**OPL**	**ONL**	**OR**
Baseline	14.63 ± 0.58	51.25 ± 1.08	25.76 ± 0.31	15.99 ± 0.49	63.30 ± 0.54	57.83 ± 1.28
1	15.77 ± 1.02	52.62 ± 1.23	27.54 ± 1.70	16.67 ± 1.12	53.63 ± 2.43	47.56 ± 1.19
3	16.21 ± 0.55	50.22 ± 1.02	33.35 ± 1.54	21.67 ± 1.97	38.08 ± 5.6	46.86 ± 2.16
6	16.08 ± 0.54	47.53 ± 0.95	32.72 ± 0.57	17.27 ± 0.59	31.60 ± 5.09	27.32↑2.25
13	15.01 ± 0.59	43.89 ± 1.47	24.99 ± 0.72	15.44 ± 1.00	22.07 ± 5.30	16.36 ± 1.65
20	15.06 ± 0.62	43.19 ± 1.02	22.52 ± 2.42	13.94 ± 1.87	16.81 ± 4.84	13.07 ± 0.68

### 3.6 Effect of SI on vitreous particles

**Figure 6:**
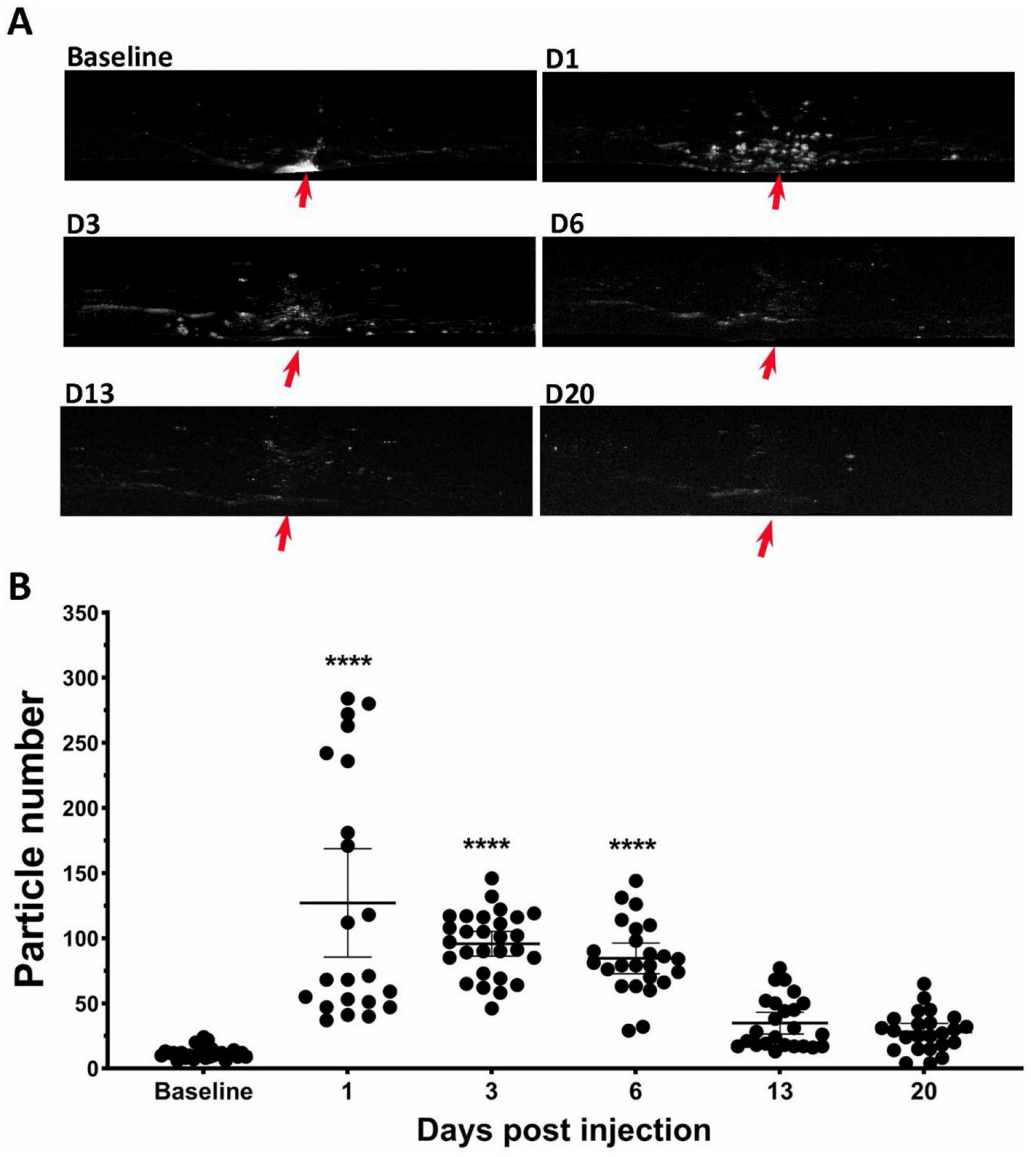
SI induced increase of particles in vitreous cavity. **(A)** Example of OCT vitreous cavity before and 1-, 3-, 6-, 13-, and 20-day post injection. Red arrows point the position of optic nerve head. **(B)** Counted particle numbers in vitreous cavity at each time points.

During OCT image acquisition, we noticed the appearance of many hyper-reflective spots floating in the vitreous cavity near the optic nerve head after mice received SI, especially on PI1 (Figure 1A). To quantify these vitreous particles, we isolated vitreous images by subtracting the retina portion from OCT images. Examples are shown in Figure 6A. After SI treatment, many small particles appear in vitreous around the vicinity of the optic nerve head (indicated by red arrows). The number of these small particles was quantified using ImageJ and results are shown in Figure 6B. There is a large increase in vitreous particles after SI injection, and the number progressively decreases with time. By PI13, the vitreous particle number is not significantly different from the baseline.

### 3.7 Effect of SI on choroid thickness

**Figure 7:**
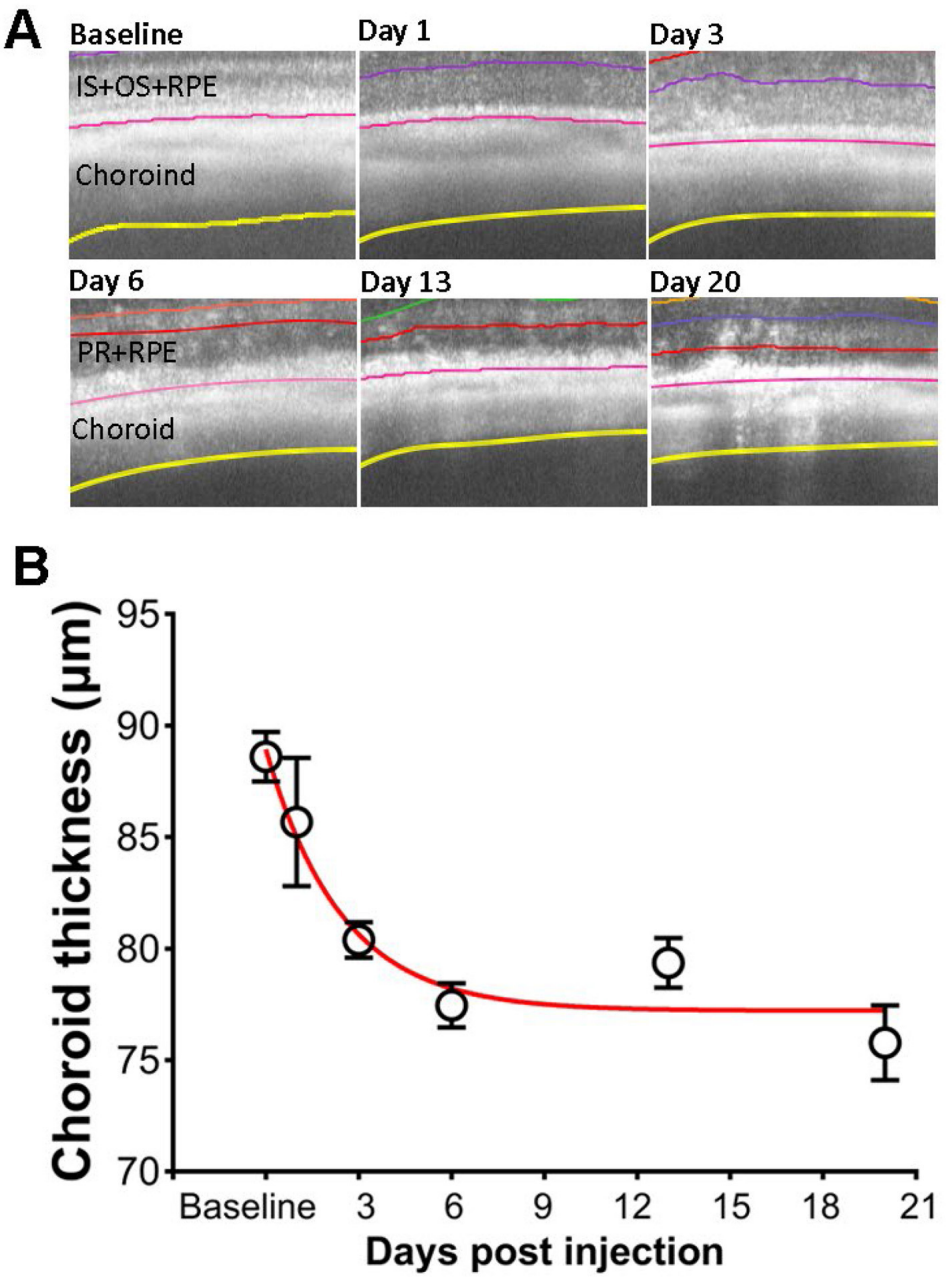
Effects of SI on choroid thickness. **(A)** Examples of OCT image with segmented choroid layer. **(B)** Choroid thickness measured at each time points.

The application of SI also induces changes in the choroid layer. Figure 7A show examples of segmented choroid layer from OCT images. The mean thickness of choroid at baseline is 88.6 ± 4.2 um (mean ± SD), similar to the published data measured by magnetic resonance imaging (MRI) (Duong, 2014). The effects of SI on choroid thickness are shown in Figure 7B. Choroid thickness showed some reduction at PI1. However, due to the large variance of the value, the difference between PI1 and baseline is not statistically significant. Choroid thickness showed further decrease with time, and all those values are significantly different from the baseline.

## 4 Discussion

Development of AI-based segmentation tools greatly facilitated analysis of OCT images. We have obtained “normal retina” models that can accurately segment images captured at baseline and PI1 and PI3 after SI injection, and “DR” models for segmenting images captured from degenerative retina (Figure 1). To check for potential bias between these two groups of models, we compared inner retinal layer thicknesses for PI3 and PI6 images. No significant differences were detected for values obtained with “normal retina” model from those obtained with “DR” model. The accurate segmentation of retinal layers enhances sensitivity to detect layer thickness changes. For example, RPE layer exhibited thickening by about 0.5 μm after SI (Figure 3A), and this change is statistically significant. Thickening of RPE layer is consistent with known action of SI specifically targeting RPE (Sorsby, 1941; Hanus et al., 2016; Hurley, 2021; Koster et al., 2022; Espitia-Arias et al., 2023; Zhang et al., 2023) to induce cell swelling and eventually death (Koh et al., 2019; Ahn et al., 2020; Hanna et al., 2022).

Retinal degeneration induces various changes in retinal structure. In our study, we found that the RPE, IS, and OS bands were merged after PI3, which might be attributed to the migration of RPE cells into the photoreceptor layer (Enzbrenner et al., 2021; Kim et al., 2022) and disorganization of photoreceptor segments (Hanus et al., 2016). We developed “degenerative retina” models to analyze OCT images with prominent photoreceptor degeneration (Figure 2B). These models were successful in processing all OCT images captures from degenerative retina. We anticipate more improvements for future models as more annotated degenerative retina images are available for new training sets.

With a combination of “normal retina” and “degenerative retina” model segmentation, we were able to characterize photoreceptor degeneration induced by SI. As shown in Figure 3C, reduction of ONL thickness can be described by a single exponential decay, with a half-decay constant of 3 days for the SI dosage used in this study (25 mg/kg). As SI-induced retinal degeneration is dose dependent (Moriguchi et al., 2018; Zhang et al., 2021), it is likely that the degeneration rate constant will also be dependent on SI dosage. We also detected an exponential reduction of choroid thickness following application of SI (Figure 7). Due to the large variation in choroid thickness at PI1, it is not possible to directly compare the time constant for choroid thickness change with ONL changes. We also investigated potential regional differences in SI-induced photoreceptor degeneration by plotting ONL and OR thickness at each of eight ROIs on the retina. As shown in Supplementary Figure 2, there is no consistent pattern for regional variation, and all 8 ROIs showed parallel ONL and OR thickness reduction. In this study, we used the mean value of 8 ROIs to represent data for each eye.

We calculated eAC from OCT images to investigate SI induced reflectance changes in retinal layers. Unlike raw OCT intensity which is influenced by many factors, including the optical quality of anterior segment, eAC is a much better measurement of tissue scattering (Vermeer et al., 2013; Chang and Bowden, 2019; Sakai et al., 2024; Bissig et al., 2025). For example, eAC is useful in revealing tissue reflectance after shadow of big blood vessels on the surface of retina. However, eAC can only be calculated from original OCT intensity on linear scale, but not from images generated by commercial instrument with modified pixel intensity (such as contrast enhancement or background subtraction). In this study, we noticed an early and persistent elevation of eAC at ONL after SI injection. On the other hand, raw OCT intensity showed slight but not statistically significant reductions (Supplementary Figure 3), suggesting there might be SI-induced optical changes in anterior chamber.

Interestingly, the level of ONL eAC observed at PI1 is highly correlated with final extent of photoreceptor degeneration at PI20 (Figure 4B). Although value of R^2^ is relatively modest (0.5), it should be noted that all mice in this study received the same dose of SI and all of them had larger than 50% retinal degeneration. The correlation exhibited a much larger *R*^2^ (0.84) with addition of a new group of mice received no SI treatment (Supplementary Figure 4). To further investigate this correlation, future studies may use different doses of SI to produce variable amount of retinal degeneration. While the mechanisms for ONL eAC elevation need further investigation, it could serve as an early biomarker for cell stress. Analyzing longitudinal OCT images from human patients and calculate ONL eAC values at various time points before and after diagnosis of retinal degeneration will be able to determine if similar correlation also exists for human subjects.

In addition to damaging the RPE and photoreceptors, we noticed that SI has impacts on all other retinal layers (Figure 5A), including the inner plexiform layer (IPL), inner nuclear layer (INL), and outer plexiform layer (OPL), but not nerve fiber layer (NFL), these layers have degeneration to different extents. Interestingly, instead of a monotonic reduction of photoreceptors, the changes in these layers are biphasic, as the thickness increased in one of the early stages, followed by degeneration at a later phase (Figure 5B). While the underlying mechanisms of inner retinal changes might be different from those of photoreceptor degeneration and need further investigation, there are reports that indicate SI-induced inflammation in the retina (Moriguchi et al., 2018; Enzbrenner et al., 2021; Zhang et al., 2021; Koster et al., 2022). SI-induced inflammation is consistent with an increased number of particles in vitreous cavity near the optic nerve head (Figure 6). Interestingly, there were large increases in the number of particles in the vitreous cavity at PI1, PI3, and PI6, the same time points when inner retinal layers showed increases in thickness (Figure 5).

Taken together, the improved AI-based segmentation tool is capable of processing OCT images from both normal and degenerative retinas. Accurate segmentation provides a reliable and sensitive measurement of OCT signals, allowing for the detection of subtle changes in degenerated retinal layers. Using this tool, we detected SI-induced swelling of RPE, reflectance increments of ONL, and transient swelling of inner retinal layer possibly induced by inflammation. Precise segmentation of OCT images provides quantitative description of retinal changes produced by degeneration, including an exponential reduction in choroid thickness. Furthermore, we have identified that ONL reflectance could serve as an early biomarker for photoreceptor degeneration.

## Data Availability

The datasets presented in this study can be found in online repositories (https://zenodo.org/), doi: 10.5281/zenodo.15054026.

## References

[B1] Ahn S. M. Ahn J. Cha S. Yun C. Park T. K. Kim Y. J.. (2020). Morphologic and electrophysiologic findings of retinal degeneration after intravitreal sodium iodate injection following vitrectomy in canines. Sci. Rep. 10:3588. 10.1038/s41598-020-60579-1 32107442 PMC7046695

[B2] Arifin W. N. Zahiruddin W. M. (2017). Sample size calculation in animal studies using resource equation approach. Malays J. Med. Sci. 24, 101–105. 10.21315/mjms2017.24.5.11 29386977 PMC5772820

[B3] Baghaie A. Yu Z. D'souza R. M. (2015). State-of-the-ar in retinal optical coherence tomography image analysis. Quant. Imaging Med. Surg. 5, 603–617. 10.3978/j.issn.2223-4292.2015.07.02 26435924 PMC4559975

[B4] Bannai D. Lizano P. Kasetty M. Lutz O. Zeng V. Sarvode S.. (2020). Retinal layer abnormalities and their association with clinical and brain measures in psychotic disorders: a preliminary study. Psychiatry Res. Neuroimaging 299:111061. 10.1016/j.pscychresns.2020.111061 32145500 PMC7183910

[B5] Berkowitz B. A. Podolsky R. H. Childers K. L. Roberts R. Katz R. Waseem R.. (2022). Transducin-deficient rod photoreceptors evaluated with optical coherence tomography and oxygen consumption rate energy biomarkers. Invest. Ophthalmol. Vis. Sci. 63:22. 10.1167/iovs.63.13.22 36576748 PMC9804021

[B6] Bissig D. Gao S. Qian H. (2025). Quantifying the influence of optical coherence tomography beam tilt in each retinal layer. PLoS One. 40493569 10.1371/journal.pone.0325217 PMC12186825

[B7] Chang S. Bowden A. K. (2019). Review of methods and applications of attenuation coefficient measurements with optical coherence tomography. J. Biomed. Opt. 24, 1–17. 10.1117/1.JBO.24.9.090901 31520468 PMC6997582

[B8] Duong T. Q. (2014). Magnetic resonance imaging of the retina: from mice to men. Magn. Reson. Med. 71, 1526–1530. 10.1002/mrm.24797 23716429 PMC3783549

[B9] Enzbrenner A. Zulliger R. Biber J. Pousa A. M. Q. Schafer N. Stucki C.. (2021). Sodium iodate-induced degeneration results in local complement changes and inflammatory processes in murine retina. Int. J. Mol. Sci. 22:9218. 10.3390/ijms22179218 34502128 PMC8431125

[B10] Espitia-Arias M. D. De La Villa P. Paleo-Garcia V. Germain F. Milla-Navarro S. (2023). Oxidative model of retinal neurodegeneration induced by sodium iodate: morphofunctional assessment of the visual pathway. Antioxidants 12:1594. 10.3390/antiox12081594 37627589 PMC10451746

[B11] Fischer M. D. Huber G. Beck S. C. Tanimoto N. Muehlfriedel R. Fahl E.. (2009). Noninvasive, in vivo assessment of mouse retinal structure using optical coherence tomography. PLoS ONE 4:e7507. 10.1371/journal.pone.0007507 19838301 PMC2759518

[B12] Furashova O. Matthe E. (2017). Retinal changes in different grades of retinal artery occlusion: an optical coherence tomography study. Invest. Ophthalmol. Vis. Sci. 58, 5209–5216. 10.1167/iovs.17-22411 29049721

[B13] Gao S. Li Y. Bissig D. Cohen E. D. Podolsky R. H. Childers K. L.. (2021). Functional regulation of an outer retina hyporeflective band on optical coherence tomography images. Sci. Rep. 11:10260. 10.1038/s41598-021-89599-1 33986362 PMC8119672

[B14] Genead M. A. Fishman G. A. Anastasakis A. (2011). Spectral-domain OCT peripapillary retinal nerve fibre layer thickness measurements in patients with Stargardt disease. Br. J. Ophthalmol. 95, 689–693. 10.1136/bjo.2010.189720 20935302 PMC3697128

[B15] Gersch J. Hufendiek K. Delarocque J. Framme C. Jacobsen C. Stohr H.. (2022). Investigation of structural alterations in inherited retinal diseases: a quantitative SD-OCT-analysis of retinal layer thicknesses in light of underlying genetic mutations. Int. J. Mol. Sci. 23:16007. 10.3390/ijms232416007 36555650 PMC9788460

[B16] Girard M. J. Strouthidis N. G. Ethier C. R. Mari J. M. (2011). Shadow removal and contrast enhancement in optical coherence tomography images of the human optic nerve head. Invest. Ophthalmol. Vis. Sci. 52, 7738–7748. 10.1167/iovs.10-6925 21551412

[B17] Hanna J. David L. A. Touahri Y. Fleming T. Screaton R. A. Schuurmans C.. (2022). Beyond genetics: the role of metabolism in photoreceptor survival, development and repair. Front. Cell. Dev. Biol. 10:887764. 10.3389/fcell.2022.887764 35663397 PMC9157592

[B18] Hanus J. Anderson C. Sarraf D. Ma J. Wang S. (2016). Retinal pigment epithelial cell necroptosis in response to sodium iodate. Cell Death Discov. 2:16054. 10.1038/cddiscovery.2016.54 27551542 PMC4979458

[B19] Hurley J. B. (2021). Retina metabolism and metabolism in the pigmented epithelium: a busy intersection. Annu. Rev. Vis. Sci. 7, 665–692. 10.1146/annurev-vision-100419-115156 34102066 PMC8922051

[B20] Keenan T. D. L. Chakravarthy U. Loewenstein A. Chew E. Y. Schmidt-Erfurth U. (2021). Automated quantitative assessment of retinal fluid volumes as important biomarkers in neovascular age-related macular degeneration. Am. J. Ophthalmol. 224, 267–281. 10.1016/j.ajo.2020.12.012 33359681 PMC8058226

[B21] Kim S. Y. Zhao Y. Kim H. L. Oh Y. Xu Q. (2022). Sodium iodate-induced retina degeneration observed in non-separate sclerochoroid/retina pigment epithelium/retina whole mounts. Ann. Eye Sci. 7:3. 10.21037/aes-21-27 36046548 PMC9427010

[B22] Koh A. E. Alsaeedi H. A. Rashid M. B. A. Lam C. Harun M. H. N. Saleh M.. (2019). Retinal degeneration rat model: A study on the structural and functional changes in the retina following injection of sodium iodate. J. Photochem. Photobiol. B. 196:111514. 10.1016/j.jphotobiol.2019.111514 31154277

[B23] Koster C. Van Den Hurk K. T. Ten Brink J. B. Lewallen C. F. Stanzel B. V. Bharti K.. (2022). Sodium-iodate injection can replicate retinal degenerative disease stages in pigmented mice and rats: non-invasive follow-up using OCT and ERG. Int. J. Mol. Sci. 23:2918. 10.3390/ijms23062918 35328338 PMC8953416

[B24] Kubo T. Akasaka T. (2010). Optical coherence tomography imaging: current status and future perspectives: current and future developments in OCT. Cardiovasc. Interv. Ther. 25, 2–10. 10.1007/s12928-009-0006-3 24122426

[B25] Kwan C. C. Fawzi A. A. (2019). Imaging and biomarkers in diabetic macular edema and diabetic retinopathy. Curr. Diab. Rep. 19:95. 10.1007/s11892-019-1226-2 31473838

[B26] Lee C. S. Tyring A. J. Deruyter N. P. Wu Y. Rokem A. Lee A. Y.. (2017). Deep-learning based, automated segmentation of macular edema in optical coherence tomography. Biomed. Opt. Express 8, 3440–3448. 10.1364/BOE.8.003440 28717579 PMC5508840

[B27] Ling K. P. Mangalesh S. Tran-Viet D. Gunther R. Toth C. A. Vajzovic L.. (2020). Handheld spectral domain optical coherence tomography findings of x-linked retinoschisis in early childhood. Retina 40, 1996–2003. 10.1097/IAE.0000000000002688 31764609 PMC8896576

[B28] Liu Y. Li Y. Wang C. Zhang Y. Su G. (2019). Morphologic and histopathologic change of sodium iodate-induced retinal degeneration in adult rats. Int. J. Clin. Exp. Pathol. 12, 443–454. 31933849 PMC6945090

[B29] Lu D. Heisler M. Lee S. Ding G. W. Navajas E. Sarunic M. V.. (2019). Deep-learning based multiclass retinal fluid segmentation and detection in optical coherence tomography images using a fully convolutional neural network. Med. Image Anal. 54, 100–110. 10.1016/j.media.2019.02.011 30856455

[B30] Mares V. Nehemy M. B. Bogunovic H. Frank S. Reiter G. S. Schmidt-Erfurth U.. (2024). AI-based support for optical coherence tomography in age-related macular degeneration. Int. J. Retina Vitreous 10:31. 10.1186/s40942-024-00549-1 38589936 PMC11000391

[B31] Moraes G. Struyven R. Wagner S. K. Liu T. Chong D. Abbas A.. (2024). Quantifying changes on OCT in eyes receiving treatment for neovascular age-related macular degeneration. Ophthalmol. Sci. 4:100570. 10.1016/j.xops.2024.100570 39224530 PMC11367487

[B32] Moriguchi M. Nakamura S. Inoue Y. Nishinaka A. Nakamura M. Shimazawa M.. (2018). Irreversible photoreceptors and RPE cells damage by intravenous sodium iodate in mice is related to macrophage accumulation. Invest. Ophthalmol. Vis. Sci. 59, 3476–3487. 10.1167/iovs.17-23532 30025075

[B33] Narendra Rao T. J. Girish G. N. Kothari A. R. Rajan J. (2019). Deep learning based sub-retinal fluid segmentation in central serous chorioretinopathy optical coherence tomography scans. Annu. Int. Conf. IEEE Eng. Med. Biol. Soc. 2019, 978–981. 10.1109/EMBC.2019.8857105 31946057

[B34] Noell W. K. (1953). Experimentally induced toxic effects on structure and function of visual cells and pigment epithelium. Am. J. Ophthalmol. 36, 103–116. 10.1016/0002-9394(53)90159-7 13050703

[B35] Noell W. K. (1954). The origin of the electroretinogram. Am. J. Ophthalmol. 38, 78–90. 10.1016/0002-9394(54)90012-4 13180621

[B36] Ou Q. Zhu T. Li P. Li Z. Wang L. Lian C.. (2018). Establishment of retinal degeneration model in rat and monkey by intravitreal injection of sodium iodate. Curr. Mol. Med. 18, 352–364. 10.2174/1566524018666181113104023 30421676

[B37] Riazi-Esfahani H. Jafari B. Azimi H. Rahimi M. Saeidian J. Pouya P.. (2024). Assessment of area and structural irregularity of retinal layers in diabetic retinopathy using machine learning and image processing techniques. Sci. Rep. 14:4013. 10.1038/s41598-024-54535-6 38369610 PMC10874958

[B38] Sakai H. Kuji R. Moriguchi Y. Yamashita S. Takamori A. Tamura M.. (2024). Optical attenuation coefficient-based en face optical coherence tomography imaging for the reliable assessment of the ellipsoid zone. J. Clin. Med. 13:7140. 10.3390/jcm13237140 39685597 PMC11642224

[B39] Schmidt-Erfurth U. Vogl W. D. Jampol L. M. Bogunovic H. (2020). Application of automated quantification of fluid volumes to anti-vegf therapy of neovascular age-related macular degeneration. Ophthalmology 127, 1211–1219. 10.1016/j.ophtha.2020.03.010 32327254

[B40] Sorsby A. (1941). Experimental pigmentary degeneration of the retina by sodium iodate. Br. J. Ophthalmol. 25, 58–62. 10.1136/bjo.25.2.58 18169746 PMC1143262

[B41] Spaide R. F. (2013). Outer retinal atrophy after regression of subretinal drusenoid deposits as a newly recognized form of late age-related macular degeneration. Retina 33, 1800–1808. 10.1097/IAE.0b013e31829c3765 23764969

[B42] Tatham A. J. Medeiros F. A. (2017). Detecting structural progression in glaucoma with optical coherence tomography. Ophthalmology 124, S57–S65. 10.1016/j.ophtha.2017.07.015 29157363 PMC6882427

[B43] Tong J. Phu J. Alonso-Caneiro D. Khuu S. K. Kalloniatis M. (2023). High sampling resolution optical coherence tomography reveals potential concurrent reductions in ganglion cell-inner plexiform and inner nuclear layer thickness but not in outer retinal thickness in glaucoma. Ophthalmic. Physiol. Opt. 43, 46–63. 10.1111/opo.13065 36416369 PMC10947055

[B44] Vermeer K. A. Mo J. Weda J. J. Lemij H. G. Boer D. e. J.F. (2013). Depth-resolved model-based reconstruction of attenuation coefficients in optical coherence tomography. Biomed. Opt. Express 5, 322–337. 10.1364/BOE.5.000322 24466497 PMC3891343

[B45] Vinekar A. Sivakumar M. Shetty R. Mahendradas P. Krishnan N. Mallipatna A.. (2010). A novel technique using spectral-domain optical coherence tomography (Spectralis, SD-OCT+HRA) to image supine non-anaesthetized infants: utility demonstrated in aggressive posterior retinopathy of prematurity. Eye 24, 379–382. 10.1038/eye.2009.313 20057510

[B46] Yang Y. C. Islam S. U. Noor A. Khan S. Afsar W. Nazir S.. (2021). Influential usage of big data and artificial intelligence in healthcare. Comput. Math. Methods Med. 2021:5812499. 10.1155/2021/5812499 34527076 PMC8437645

[B47] Zeng Y. Gao S. Li Y. Marangoni D. Silva D. e. Wong T.. (2024). OCT Intensity of the region between outer retina band 2 and band 3 as a biomarker for retinal degeneration and therapy. Bioengineering 11:449. 10.3390/bioengineering11050449 38790316 PMC11118669

[B48] Zeng Y. Petralia R. S. Vijayasarathy C. Wu Z. Hiriyanna S. Song H.. (2016). Retinal Structure and gene therapy outcome in retinoschisin-deficient mice assessed by spectral-domain optical coherence tomography. Invest. Ophthalmol. Vis. Sci. 57, OCT277–287. 10.1167/iovs.15-18920 27409484 PMC4968785

[B49] Zhang N. Zhang X. Girardot P. E. Chrenek M. A. Sellers J. T. Li Y.. (2021). Electrophysiologic and morphologic strain differences in a low-dose NaIO_3_-induced retinal pigment epithelium damage model. Transl. Vis. Sci. Technol. 10:10. 10.1167/tvst.10.8.10 34251426 PMC8287050

[B50] Zhang S. M. Fan B. Li Y. L. Zuo Z. Y. Li G. Y. (2023). Oxidative stress-involved mitophagy of retinal pigment epithelium and retinal degenerative diseases. Cell Mol. Neurobiol. 43, 3265–3276. 10.1007/s10571-023-01383-z 37391574 PMC10477140

