# Supplementary material for: Evaluation of retinal structure changes with AI-based OCT image segmentation for sodium iodate induced retinal degeneration
Source: Front Cell Neurosci. 2025 Jun 18;19:1605639. doi: 10.3389/fncel.2025.1605639 (PMC12213626; doi:10.3389/fncel.2025.1605639)
Supplement: Supplementary file 1 [file Data_Sheet_1.docx]

| **Model** | **Line1** | | **Line2** | | **Line3** | | **Line4** | | **Line5** | | **Line6** | | **Line7** | | **Line8** | | **Line9** | | **Sum** |
| --- | --- | --- | --- | --- | --- | --- | --- | --- | --- | --- | --- | --- | --- | --- | --- | --- | --- | --- | --- |
|  | **L** | **R** | **L** | **R** | **L** | **R** | **L** | **R** | **L** | **R** | **L** | **R** | **L** | **R** | **L** | **R** | **L** | **R** |  |
| **1** | 33 | 32 | 29 | 32 | 11 | 9 | 28 | 30 | 32 | 20 | 4 | 7 | 43 | 54 | 0 | 0 | 49 | 31 | 444 |
| **2** | 15 | 19 | 34 | 31 | 18 | 12 | 18 | 20 | 41 | 28 | 46 | 28 | 15 | 9 | 0 | 0 | 43 | 4 | 381 |
| **3** | 22 | 24 | 29 | 35 | 32 | 25 | 16 | 27 | 36 | 27 | 46 | 32 | 27 | 30 | 0 | 0 | 36 | 19 | 463 |
| **4** | 30 | 31 | 30 | 25 | 32 | 28 | 10 | 24 | 23 | 31 | 17 | 27 | 143 | 107 | 0 | 0 | 8 | 7 | 573 |
| **5** | 69 | 57 | 42 | 42 | 62 | 96 | 83 | 66 | 28 | 18 | 33 | 26 | 24 | 51 | 0 | 0 | 4 | 1 | 702 |
| **6** | 22 | 21 | 33 | 37 | 34 | 35 | 37 | 36 | 27 | 29 | 74 | 96 | 8 | 12 | 0 | 0 | 4 | 2 | 507 |
| **7** | 37 | 18 | 20 | 27 | 42 | 30 | 17 | 15 | 26 | 32 | 24 | 36 | 16 | 16 | 0 | 0 | 30 | 39 | 425 |
| **8** | 26 | 22 | 30 | 28 | 18 | 32 | 47 | 46 | 48 | 48 | 14 | 16 | 9 | 9 | 0 | 0 | 9 | 25 | 427 |
| **9** | 27 | 46 | 27 | 12 | 19 | 10 | 21 | 13 | 21 | 54 | 32 | 25 | 20 | 11 | 0 | 0 | 71 | 52 | 461 |
| **10** | 25 | 37 | 30 | 38 | 30 | 19 | 39 | 35 | 32 | 25 | 10 | 6 | 5 | 6 | 0 | 0 | 29 | 49 | 415 |
| **Model-Median** | 30 | 29 | 32 | 29 | 38 | 40 | 17 | 22 | 19 | 22 | 36 | 37 | 26 | 31 | 336 | 336 | 53 | 107 | 1240 |
| **Corrected** | 0 | 0 | 0 | 0 | 0 | 0 | 3 | 2 | 3 | 2 | 0 | 0 | 0 | 0 | 0 | 0 | 0 | 0 | 10 |

Supplementary Material

Supplemental Table 1: Usage rate of normal retina models

Supplemental Table 2: DICE score of normal retina models

| **Model** | **Mean** | **SD** |
| --- | --- | --- |
| **1** | 0.973 | 0.020 |
| **2** | 0.956 | 0.038 |
| **3** | 0.972 | 0.022 |
| **4** | 0.975 | 0.019 |
| **5** | 0.972 | 0.021 |
| **6** | 0.973 | 0.025 |
| **7** | 0.973 | 0.021 |
| **8** | 0.977 | 0.016 |
| **9** | 0.969 | 0.024 |
| **10** | 0.925 | 0.039 |

Supplemental Table 3: Usage rate of degenerative retina models

| **Model** | **Line1** | | **Line2** | | **Line3** | | **Line4** | | **Line5** | | **Line6** | | **Line7** | | **Line8** | | **Sum** |
| --- | --- | --- | --- | --- | --- | --- | --- | --- | --- | --- | --- | --- | --- | --- | --- | --- | --- |
|  | **L** | **R** | **L** | **R** | **L** | **R** | **L** | **R** | **L** | **R** | **L** | **R** | **L** | **R** | **L** | **R** |  |
| **DR1** | 0 | 1 | 0 | 0 | 0 | 1 | 19 | 12 | 2 | 0 | 0 | 0 | 0 | 0 | 24 | 32 | 91 |
| **DR2** | 0 | 0 | 0 | 0 | 0 | 2 | 9 | 2 | 0 | 1 | 2 | 2 | 0 | 0 | 54 | 47 | 119 |
| **DR3** | 1 | 0 | 0 | 1 | 0 | 0 | 5 | 1 | 3 | 1 | 22 | 26 | 0 | 1 | 92 | 101 | 254 |
| **DR4** | 0 | 0 | 1 | 0 | 1 | 0 | 2 | 0 | 0 | 0 | 2 | 1 | 1 | 0 | 6 | 8 | 22 |
| **DR5** | 1 | 1 | 1 | 0 | 1 | 0 | 19 | 18 | 6 | 4 | 0 | 2 | 0 | 0 | 1 | 1 | 55 |
| **DR6** | 0 | 0 | 0 | 0 | 0 | 0 | 2 | 2 | 1 | 0 | 0 | 1 | 0 | 0 | 0 | 0 | 6 |
| **DR7** | 0 | 0 | 1 | 0 | 1 | 1 | 6 | 2 | 0 | 1 | 64 | 60 | 1 | 0 | 0 | 1 | 138 |
| **DR8** | 0 | 0 | 0 | 0 | 0 | 0 | 5 | 9 | 4 | 2 | 0 | 0 | 0 | 0 | 0 | 0 | 20 |
| **DR9** | 2 | 1 | 0 | 0 | 1 | 3 | 58 | 56 | 2 | 6 | 2 | 2 | 0 | 0 | 1 | 2 | 136 |
| **DR10** | 0 | 0 | 0 | 2 | 0 | 0 | 13 | 20 | 1 | 0 | 0 | 0 | 0 | 2 | 0 | 0 | 38 |
| **DR11** | 0 | 1 | 0 | 1 | 1 | 2 | 1 | 2 | 0 | 0 | 1 | 2 | 0 | 0 | 0 | 1 | 12 |
| **Median** | 274 | 274 | 275 | 274 | 273 | 269 | 139 | 154 | 259 | 263 | 185 | 182 | 276 | 275 | 100 | 85 | 3557 |

Supplemental Table 4: DICE score of degenerative retina models

| **Model** | **Mean** | **SD** |
| --- | --- | --- |
| **DR1** | 0.992 | 0.004 |
| **DR2** | 0.991 | 0.005 |
| **DR3** | 0.992 | 0.005 |
| **DR4** | 0.991 | 0.005 |
| **DR5** | 0.991 | 0.004 |
| **DR6** | 0.992 | 0.004 |
| **DR7** | 0.992 | 0.004 |
| **DR8** | 0.992 | 0.005 |
| **DR9** | 0.992 | 0.004 |
| **DR10** | 0.991 | 0.004 |
| **DR11** | 0.982 | 0.009 |


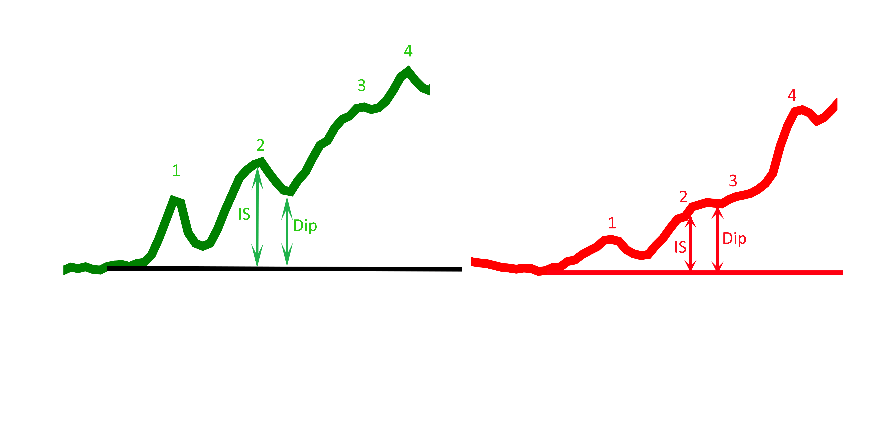


Supplemental Figure S1, Illustration of Dip ratio calculation. The curves plot OCT intensity profile of outer retinal band (1: ELM; 2: IS; 3: OS; 4: RPE) from images captured at baseline (left, green) and at PI3 (right, red). Double-head arrows label OCT intensity at peak of IS band and intensity at Dip region. Dip ratio is defined as OCT intensity at IS peak divided by the value at Dip region.


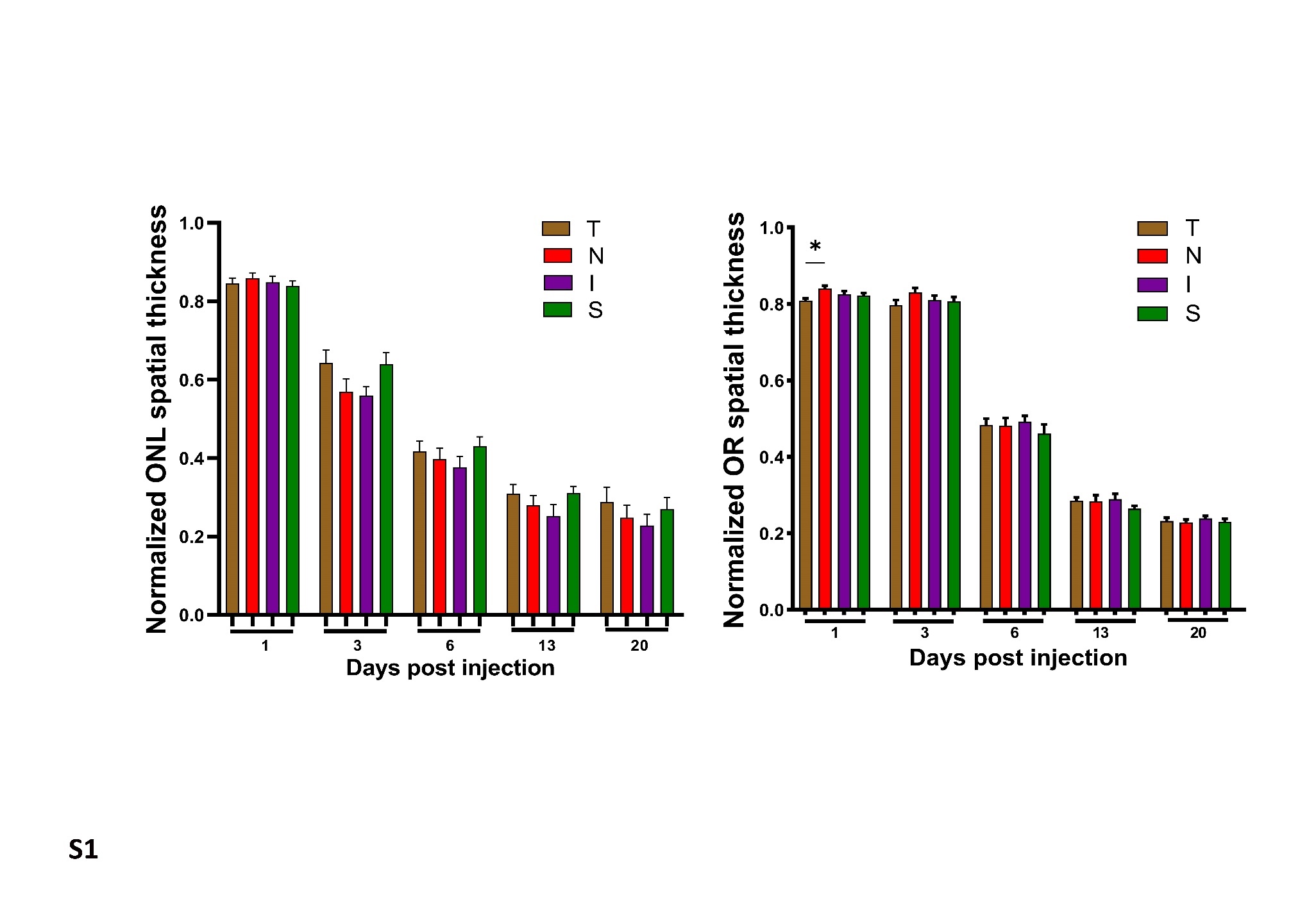


Supplemental Figure S2, Normalized layer thickness changes measured at 4 retinal regions. T: temporal retina; N: nasal retina; I: inferior retina; S: superior retina.


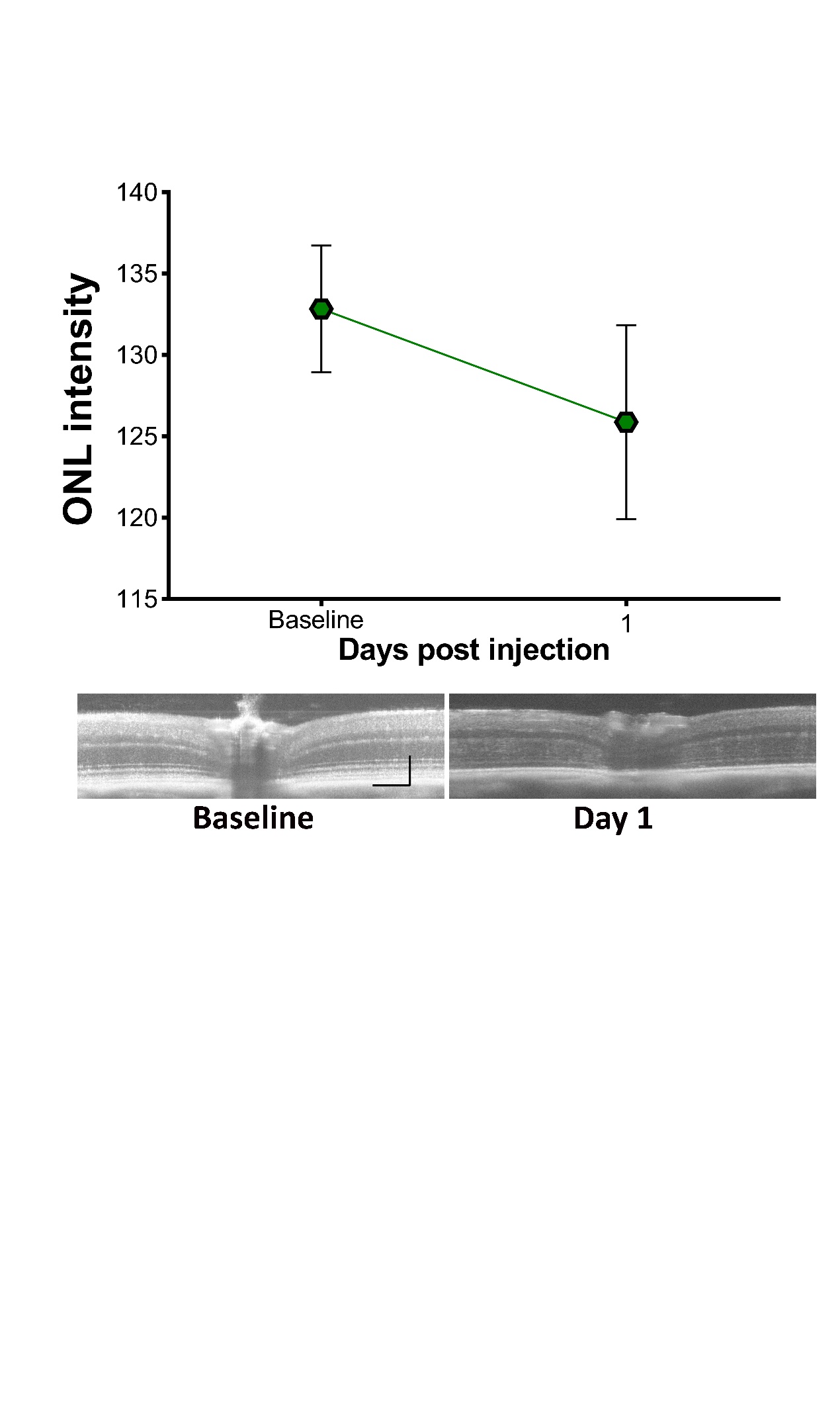


Supplemental Figure S3, OCT intensity of ONL calculated from baseline and PI1 images, with example OCT image shown at bottom.


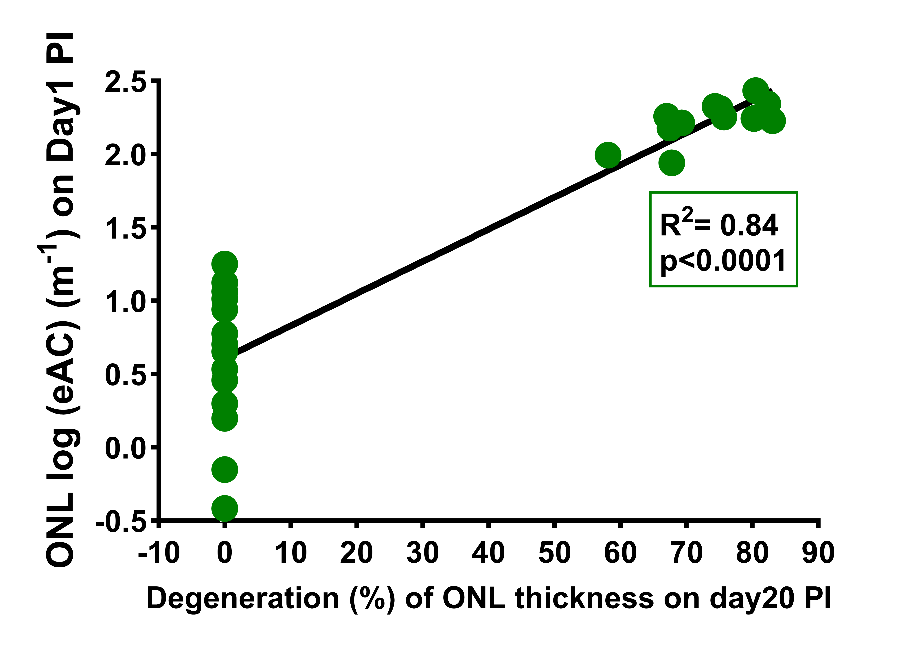


Supplemental Figure S4, Correlation between photoreceptor degeneration (ONL reduction at PI20) with ONL eAC values at PI1. Data were pooled from two groups of mice, one group received 25 mg/kg SI (data shown in Fig. 4B) and the other group received no SI.
